# Thyroxine. Osteoporosis and Fractures in the Mentally Handicapped

**Published:** 1990-03

**Authors:** J. Jancar

**Affiliations:** Honorary Consultant Psychiatrist, Stoke Park Hospital, Stapleton, Bristol BS16 1QU


### Thyroxine, Osteoporosis and Fractures in the Mentally Handicapped

**J. Jancar, MB, BCh, BAO, FRCPsych, DPM**
Honorary Consultant Psychiatrist, Stoke Park Hospital, Stapleton, Bristol BS16 1QU

### INTRODUCTION

Since von Recklinghausen reported in 1891 severe skeletal demineralization in a 23 year old woman with thyrotoxicosis (1), numerous studies have reported the abnormal bone and mineral dynamics in hyperthyroidism.

Similarly, a large number of patients may be at risk for iatrogenic bone demineralization for exogenous thyroid hormone, given either for treatment of hypothyroidism or as suppressive therapy for thyroid neoplasias. Several studies indicate that patients taking thyroid hormone for well-accepted indications, and at the dosages that do not cause clinical hyperthyroidism, are at risk of developing osteoporosis (2, 3).

In view of these latest studies relating to the normal population, in September 1988 we examined the case notes of all the patients receiving thyroxine therapy in the Stoke Park Group of Hospitals in Bristol which housed 682 (377 male and 305 female) mostly adult and elderly severely mentally handicapped patients.

### RESULTS

Fourteen females (4.6%) were on thyroxine treatment for hypothyroidism. Ages ranged from 42 to 89 years (average 63.3 years). They had been on treatment for between three and 25 years (average 11.4 years). The dosage varied from 50–150 micrograms daily. Average IQ was 28.3 and Mental Age 4.0. Among 14 females who were on thyroxine, there were two with Down's syndrome, two "cretins", one post-thyroidectomy patient and two epileptics.

Four female patients suffered from osteoporosis. Five other female patients sustained fractures, one of which was a fracture of the neck of the femur, but osteoporosis was not recorded in any of their case-notes.

Five males (1.3%) were being treated with thyroxine for hypothyroidism. Their ages ranged from 44 to 62 years (average 54 years). They had been receiving treatment for between two and 32 years (average 11.2 years). The daily dosages of thyroxine ranged from 100–200 micrograms. Average IQ was 25.4 and Mental Age 4.0.

In this group of patients there were three with Down's syndrome and two epileptics. Osteoporosis was not recorded in the case-notes of these patients but one sustained a fracture.

### CONCLUSION

Although there are other causes of osteoporosis and fractures in the mentally handicapped (4), nevertheless it is important that the dosage of thyroxine is constantly monitored and adjustments made according to the results of assays. Patients should be regularly checked for early signs of osteoporosis, particularly older women whose skeletons are most at risk.

Because of increased longevity of the mentally handicapped population, cases of hypothyroidism are likely to increase, particularly in patients with Down's syndrome (5). Reports from the normal population and the results of our survey of a selective sample of 19 hospitalized mentally handicapped patients indicate the need for further study of the effects and side-effects of thyroxine therapy in the mentally handicapped population.

### REFERENCES

1. VON RECKLINGHAUSEN F (1891). Die fibrose oder deformirende Ostitis die Osteomalacie und die Osteoplastische Carcinose, in ihren gegenseitigen Bezichungen. Festschrift Rudolf Virchow, Berlin: George Reimer, 1–89.
2. COOPER D. S. (1988). Thyroid Hormone and the Skeleton: A Bone of contention. *JAMA*, **259**, 3175.
3. PAUL T. L., KERRIGAN J., KELLY ANN MARIE, BRAVERMAN L. E. and BARAN D. T. (1988). Long-term L-Thyroxine Therapy is associated with decreased Hip Bone Density in Pre-menopausal Women. *JAMA*, **259**, 3137–41.
4. JANCAR J. (1988). Consequences of longer life for the mentally handicapped. *Geriatric Medicine*, 81–7.
5. MANI C. (1988). Hypothyroidism in Down's syndrome. *Brit. J. Psychiat,* **153**, 102–4.

